# Pericardectomy after pericarditis constrictiva led to onset of transplant kidney function after 98 days of anuric kidney graft: a case report

**DOI:** 10.1186/s12882-020-01899-2

**Published:** 2020-06-29

**Authors:** Caroline Wacker, Michael Weyand, Mario Schiffer, Mirian Opgenoorth

**Affiliations:** 1 Department of Nephrology and Hypertension, University Erlangen-Nürnberg, Ulmenweg 18, 91054 Erlangen, Germany; 2 Department of Cardiac Surgery, University Erlangen-Nürnberg, Krankenhausstraße 12, 91054 Erlangen, Germany

**Keywords:** Constrictive pericarditis, Kidney transplant, Delayed graft function, Pericardectomy, Case report

## Abstract

**Background:**

Constrictive pericarditis is easily overlooked and can lead to severe problems in hemodynamics and end-organ perfusion, in our patient leading to 98 days of anuria after living kidney transplantation. This was completely reversible after pericardectomy.

**Case presentation:**

A 43-year-old female caucasian patient received a living kidney donation from her mother. She had developed end-stage renal disease 2 years prior due to nephrotic syndrome linked to graft-versus-host disease after allogenic stem-cell transplantation for aplastic anemia.

The graft showed insufficient function already in the early postoperative phase. Dialysis was paused after surgery, but the patient developed hypervolemia with ascites and edema in the lower extremities. Doppler ultrasonography showed scarce perfusion, with intrarenal arterial waveforms without end-diastolic flow. The venous perfusion profiles showed pulsatile retrograde flow. There was no identifiable reason for a primary vascular perfusion problem on ultrasonography or transplant kidney angiography. Kidney transplant biopsy revealed no rejection but extensive acute tubular necrosis. Three weeks after transplantation, the patient developed an acute anuric graft failure caused by severe cardiac decompensation. Echocardiography revealed a previously unnoticed constrictive pericarditis, which could be confirmed in a cardio computed tomography scan. The constrictive pericarditis had not been apparent on previous x-rays, computed tomography scans, or echocardiographies, including those for transplantation evaluation.

Conservative management of the constrictive pericarditis was not successful and the graft remained anuric. Eventually, the patient underwent pericardectomy 16 weeks after kidney transplantation. Shortly after surgery, the graft started urine production again, which significantly increased within a few days. The clearance improved and 2 weeks later, the patient was free from dialysis.

**Conclusions:**

This case illustrates that special attention should be given to the pericardium during transplant evaluation, especially for patients who previously underwent stem-cell transplantations, chemotherapy or radiation.

## Background

Constrictive pericarditis (CP) is hard to diagnose in echocardiography and often overlooked. Untreated, it can lead to severe problems in hemodynamic regulation and end-organ perfusion, which can mean a decreased transplant function, as demonstrated in this case.

Up to now, only a few case reports have been published on cases of constrictive pericarditis in kidney transplant patients. These include a case in Sri Lanka where pericardiocentesis had led to onset of graft function in the early phase on the second day after transplantation [1], and a Polish patient who developed CP a year after transplantation and displayed impaired graft function, which could be resolved by pericardectomy [2]. Four more cases of pericardectomy for CP several months to years after transplantation are described in [3] and [4]. All cases had a good prognosis after therapy.

Unique for our case is the long phase of complete anuria (98 days), which required dialysis treatment and was followed by a complete recovery of kidney function after pericardectomy; in all previously described cases, the anuric phase only lasted up to 2 days.

## Case presentation

A 43-year-old female caucasian patient received a living kidney donation from her mother. Two and a half years before, the patient had received high dose chemotherapy and an allogenic stem-cell transplantation due to aplastic anemia and secondary acute myeloic leukemia. Following this, the patient developed chronic graft-versus-host disease and, due to this, severe acute kidney failure with nephrotic syndrome and end-stage renal disease. Regular dialysis treatment was started 6 months before the living kidney donation.

Pre-transplant evaluations (including chest x-ray, ECG and echocardiogram) were unremarkable; the only issues were small amounts of ascites and a slightly dilated inferior vena cava (1.8 cm) without inspirational collapse in sonography. Both sonographic findings were attributed to hypervolemic phases in between dialysis treatment. Hypervolemia was also visible in dilated jugular veins.

Tuberculosis was ruled out during evaluation via chest x-ray and negative quantiferone test.

Donor nephrectomy and kidney transplantation were performed without surgical complications. The patient showed low immunological risk, i.e. there was no evidence of preformed HLA antibodies, especially no donor specific antibodies. Mismatch was 0–1-0. She was treated with tacrolimus, mycophenolate mofetil and prednisolone and received induction with basiliximab; moreover, she received CMV prophylaxis with valganciclovir and *Pneumocystis jirovecii* prophylaxis.

After kidney transplantation, dialysis was paused. Urinary output in the first 24 h was only 600 ml and remained similar in extent over the next days. Within days, the patient developed hypervolemia with ascites and edema in the lower extremities and high BNP levels (max. 17,395 pg/ml). Serum creatinine remained markedly increased at 3,5 mg/dl, which – in the first place – was attributed to high tacrolimus levels (max. 15 ng/ml). Blood pressure was around 100–110/70 mmHg (measured three times a day) throughout the patient’s stay.

Doppler ultrasonography showed homogeneous perfusion without end-diastolic flow. The venous perfusion profiles showed pulsatile retrograde flow. There was no identifiable reason for a primary vascular perfusion problem on ultrasonography, particularly no sign of renal vein thrombosis; subsequent transplant kidney angiography also did not find any reason.

Urine analysis was performed once to twice a week; the only findings were intermittent slight eumorphic erythrocyturia, which was attributed to the in-situ ureter stent.

At first, echocardiography did not reveal any reason for the volume overload, but showed normal ejection fraction.

**Fig. 1:**
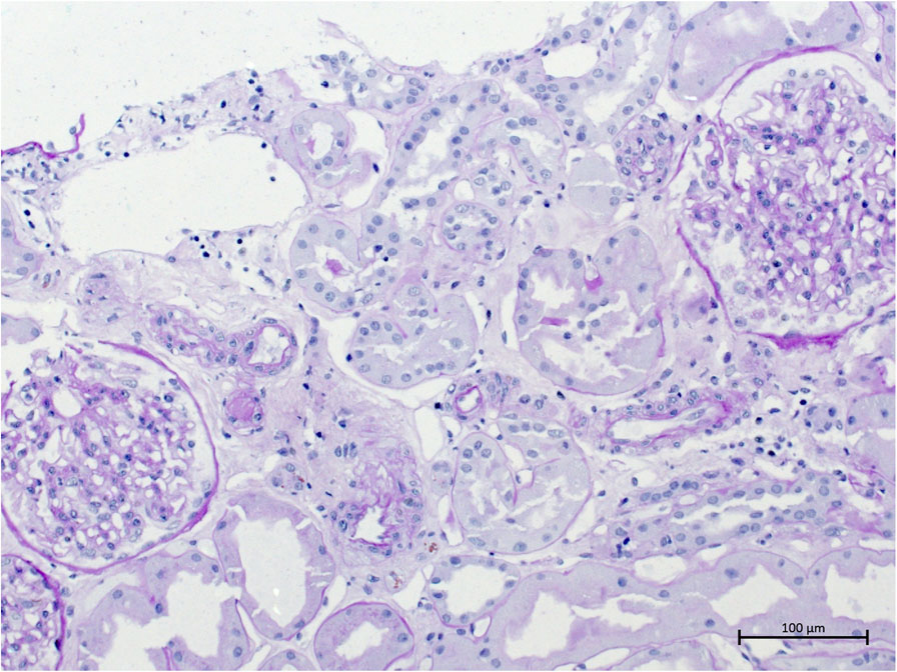
This figure shows a part of the patient’s renal cortex, displaying acute tubular necrosis while glomeruli are intact

**Fig. 2:**
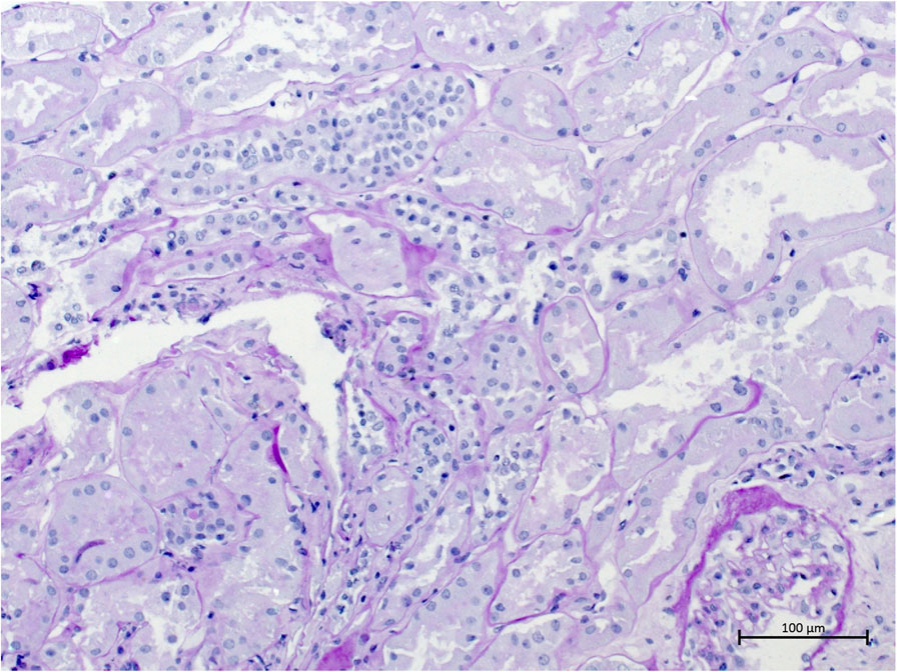
This image of the patient’s renal cortex displays signs of acute tubular necrosis as well (early phase with dense tubuli, but also wide tubuli)

Drinking restriction and lowering of tacrolimus levels to 5 ng/ml led to a slow decrease in serum creatinine (minimum 2,49 mg/dl). Three weeks after transplantation, when serum creatinine stagnated and kidney perfusion still showed end-diastolic no-flow, volume excess was tried and the patient received three liters of intravenuous fluids in order to see if perfusion improved. This led to the development of an acute anuric graft failure, most likely caused by severe cardiac decompensation. Kidney biopsy was performed in order to rule out graft rejection; the report revealed only extensive acute tubular necrosis (see Figs. 1 and 2). Dialysis was started again.

Another echocardiography revealed normal right- and leftventricular function, an ejection fraction around 70%, but distinct septal bounce phenomenon and a pronounced respiratory variation in mitral inflow velocity (30%), as often seen in constrictive pericarditis. This diagnosis was confirmed via cardio CT scan.

A conservative approach with optimal volume management and daily hemodialysis was tried for the constrictive pericarditis.

At last, a change in immunosuppressive therapy free from calcineurin inhibitors was decided and the patient received a belatacept-based medication.

**Fig. 3:**
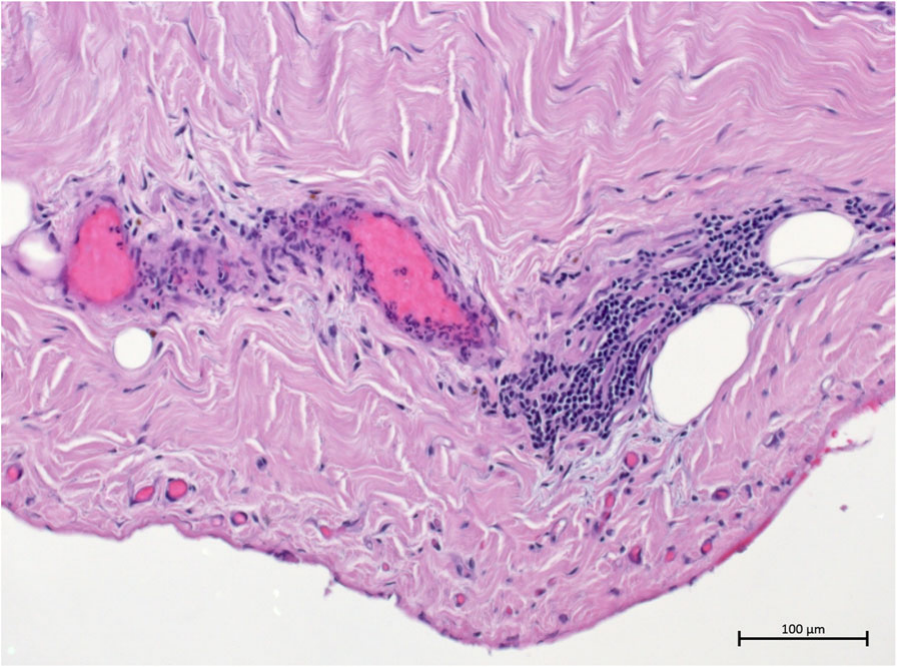
In this part of the pericardium biopsy, fibrosis can be seen as well as invasion of inflammatory cells as a manifestation of pericarditis

**Fig. 4:**
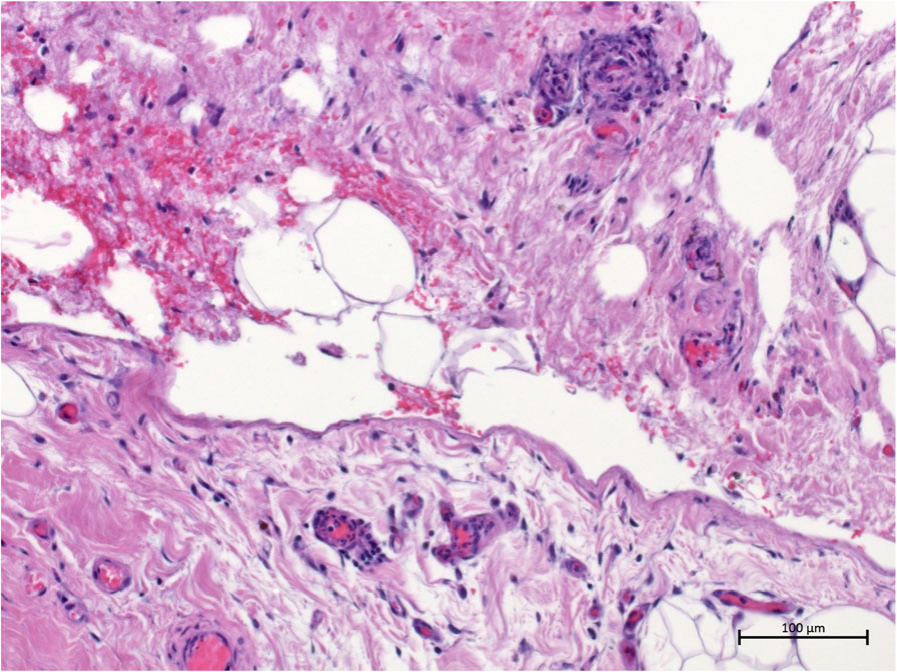
The displayed part of the pericardium consists of even more severe sclerosis, contributing to the constrictive part of the pericarditis, and also depicts angiectasias.

Despite all these actions, the graft remained anuric. Eventually, the patient underwent pericardectomy 16 weeks after kidney transplantation (13 weeks after beginning of anuric phase). Histology of the obtained pericardium was compatible with constrictive pericarditis, showing severely sclerosed pericardium with angiectasias and focal granulocyte infiltration (see Figs. 3 and 4). Within hours after surgery, the graft started urine production again; this significantly increased within days. Intermittently, the patient even showed polyuria. Glomerular filtration rate (GFR) also improved; 2 weeks later, the patient was – after 98 day of anuria – free from dialysis.

In the following months, serum creatine levels decreased even further with a serum creatinine of 1,37 mg/dl at its lowest (GFR 47 ml/min).

## Discussion and conclusions

In constrictive pericarditis, cardiac filling is impeded by the inelastic pericardium, which leads to an impaired ability to adapt to volume changes. This often results in symptoms related to hypervolemia such as peripheral edema/anasarca and/or symptoms related to diminished cardiac output, such as fatigability or dyspnea. In our case, similar to what the authors described in [3], the constrictive pericarditis and its symptoms were masked by dialysis treatment and only came to full impact when dialysis was stopped.

Reasons for development of constrictive pericarditis are manifold. Leading causes, apart from idiopathic CP and post-cardiac surgery, include post-radiation therapy and infectious etiologies [5–8]. Also, cases associated with chronic graft-versus-host-disease [9, 10] and chemotherapy [11, 12] are described, one or more of which might be the cause of CP in our patient.

Diagnosis of CP is often challenging. Electrocardiography is often unobtrusive, with sometimes nonspecific ST and T wave changes and tachycardia or low voltage present [13]. Chest radiograph might show signs for pericardial effusion or, in some cases, even pericardial calcification [13]; however, our patient did not display suspicious findings in both. While there is no single diagnostic echocardiographic parameter, there are a number of suggestive criteria such as presence of increased pericardial thickness, abnormal septal motion, pronounced respiratory variation in ventricular filling, bi-atrial enlargement, and dilated inferior vena cava and hepatic vein, typically in combination [13]. This requires an experienced echocardiographer; in retrospect, some of the clues may already have been present pre-transplant and at the first echocardiogram but were overlooked.

Our patient was lucky to experience a full resolution of symptoms even after delayed diagnosis and prolonged time until pericardectomy, but this may not always be the case. The take-away message is to take constrictive pericarditis into consideration in patients at risk (as described above) and with symptoms of right-sided heart failure, seek establishment of definitive diagnosis by echocardiography and cardiac computed tomography, cardiac magnetic resonance imaging or catheterization and eventually initiate treatment as early as possible.

On the bright side: While long-term delayed graft function often is highly associated with detrimental impact on graft function and survival [14, 15], our case gives hope concerning recovery, especially in this severe case with anuria over several weeks. This is not only true for allograft kidneys but for kidney function in general.

There have been rare reports of complete recovery of kidney function after long periods of anuria, including a period of 14 anuric days in a case of acute suprarenal occlusion [16], a period of 72 days after ureteral obstruction [17] and even a period of 315 days in a heart-transplanted 15-year old with hypovolemia [18]. However, our case with 98 days of anuria is the first of such a long period reported in a kidney allograft and also the first in a patient with constrictive pericarditis.

## Methods

For capturing the microscopy images, an Imager A2 microscope with Axiocam Icc5 (Zeiss, Germany) was used. The acquisition software was ZEN 2.6 (Zeiss, Germany). Images were acquired at 200x magnification with a resolution of 300 dpi (1388 × 1040 pixels).

## Data Availability

The datasets used during the current study are available from the corresponding author on reasonable request.

## Abbreviations

CP: Constrictive Pericarditis
CT: Computed Tomography
GFR: Glomerular Filtration Rate
Pro-BNP: Pro-brain natriuretic peptide
